# Building envelope materials, indoor environmental quality and public-health relevance in Kuwait: a narrative review of exposure pathways in a hot-arid climate

**DOI:** 10.3389/fpubh.2026.1982898

**Published:** 2026-10-26

**Authors:** Mohamad Al Hallak, Mohamad Ali-Ahmad, Christina El-Sawda, Hassan Flity, Farjallah Alassaad

**Affiliations:** College of Engineering and Technology, American University of the Middle East, Egaila, Kuwait

**Keywords:** building-envelope, exposure pathways, hot-arid conditions, indoor air quality, indoor environmental quality, thermal insulation

## Abstract

Kuwait’s hot-arid climate, intense solar exposure, dust conditions, and prolonged reliance on air-conditioning create distinctive demands on building-envelope materials and may influence their long-term interaction with indoor environments. This narrative review examines building-envelope and insulation materials investigated in Kuwait or under Kuwait climatic conditions and evaluates their potential indoor environmental and public-health relevance through measurable exposure pathways. Kuwait-focused evidence was identified primarily through Scopus and complemented by international literature where direct Kuwait-specific exposure or health evidence was unavailable. The current review is a source-pathway-measurement synthesis that relates envelope assemblies and transport mechanisms with measurable indoor environmental indicators without drawing Kuwait-specific health conclusions. Environmental and operational factors, including high surface temperatures, solar exposure, dust deposition, thermal cycling, prolonged air-conditioning, moisture gradients, installation, maintenance, and aging, may modify material behavior and the conditions under which exposure pathways become relevant. Accordingly, public-health relevance should be assessed through measurable indicators source-release-transport pathways rather than inferred from material type alone. Additionally, simulation or portable-cabin findings should not be considered functionally similar to occupied-building exposure evidence. Therefore, this review proposes an integrated assembly-transport-IEQ-occupant framework combining thermal performance, material condition and aging, ventilation and moisture assessment, targeted chemical or particulate measurements where relevant pathway exists, and occupant-reported Indoor Environmental Quality (IEQ). This structured approach underlines the measurements needed to assess the public-health relevance of building-envelope materials under Kuwait’s hot-arid and highly air-conditioned conditions.

## 1 Introduction

Kuwait presents a demanding context for building-envelope design because high outdoor temperatures, intense solar radiation, dust exposure, and long cooling periods increase the dependence of buildings on air-conditioning. In this context, roofs, façades, walls, glazing systems, insulation layers, coatings, and other envelope components play an important role in controlling solar heat gain, indoor thermal stability, and cooling-energy demand. Earlier Kuwait-based studies showed that residential energy consumption is strongly influenced by building characteristics, occupant operation, air-conditioning schedules, and thermostat settings (1, 2). More recent work has further emphasized the importance of building-envelope design, window-to-wall ratio, orientation, cooling operation, and residential building archetypes in Kuwait and the wider Gulf region (3–5). Additional Kuwait and regional studies on indoor thermal conditions, PMV-based HVAC control, data-driven building performance, energy retrofitting, and cooling-dominated insulation and glazing further support the need to consider envelope performance together with building operation and comfort (6–10).

Within this context, Kuwait-based research has examined several thermal insulation and building-envelope strategies to reduce cooling loads and improve energy performance. These include phase change materials (PCMs), Bio-PCMs, polyurethane sandwich panels, conventional insulation layers, reflective and cool-roof systems, solar-control films, smart-window technologies, and thermally efficient walling materials. PCMs represent one of the most frequently investigated groups, with studies focusing on melting temperature, material location, wall and roof integration, envelope orientation, and daily thermal cycling under Kuwait or Kuwait-like climatic conditions (11–13). International reviews on PCM adaptation, microencapsulation durability, thermal reliability, and dynamic-insulation integration also support the need to consider long-term material behavior in envelope applications (14–17).

Although these studies provide useful evidence on energy performance, the indoor environmental and public-health relevance of insulation and envelope materials remains less directly addressed. Most Kuwait studies focus on cooling-load reduction, heat-gain control, indoor temperature moderation, and energy savings, while fewer studies examine material aging, ventilation interaction, moisture movement, chemical indicators, odor perception, dust, fibers, particulate indicators, or occupant-related indoor environmental responses. This gap does not imply that the materials are unsuitable or unsafe. Rather, it identifies an evidence gap between the relatively well-developed assessment of thermal and energy performance and the much more limited evaluation of indoor environmental quality, exposure pathways, and occupant-related public health indicators within the hot-arid weather and highly air-conditioned buildings in Kuwait.

The public-health relevance of building-envelope materials should be understood through exposure pathways rather than through material type alone. Material formulation, aging, thermal cycling, moisture conditions, physical disturbance, ventilation, and maintenance can influence whether material-related indicators become relevant to occupied indoor spaces. International literature shows that temperature and humidity can influence volatile organic compound emissions from building materials, that dust and fibers are often most relevant during installation or disturbance, and that persistent dampness may be associated with indoor environmental conditions of public-health interest (18–20).

Unlike previous Kuwait-focused investigations that have predominantly evaluated building-envelope systems through thermal performance and cooling-energy outcomes, this review adopts an integrated indoor environmental and public-health perspective. It first identifies the insulation and envelope materials investigated in Kuwait or under Kuwait climatic conditions, then examines the climatic and operational factors that may modify their behavior over time, and finally evaluates the measurable indoor environmental indicators and exposure pathways through which these materials and assemblies may become relevant to occupants. International evidence is used to interpret these pathways where direct Kuwait-specific exposure or health evidence is unavailable, without inferring Kuwait-specific health effects. The originality of this review lies in applying a source–release–transport–measurement framework, in which a material is considered relevant to indoor exposure only when a reasonable route to the occupied space and a measurable indicator can be defined.

Accordingly, this review addresses three questions:

(1) Which insulation and building-envelope materials have been investigated in Kuwait or under Kuwait climatic conditions?
(2) Which environmental and operational factors may influence their long-term behavior and interaction with indoor spaces? and
(3) Which measurable indoor environmental indicators and exposure pathways should be prioritized when assessing their potential public-health relevance?

Figure 1 illustrates the conceptual framework followed to organize this study. It presents how Kuwait’s environmental and operational condition may impact envelope materials, how the later may interact with the indoor environment, and how related indicators can be linked to indoor environmental quality and occupant-related considerations.

**Figure 1:**
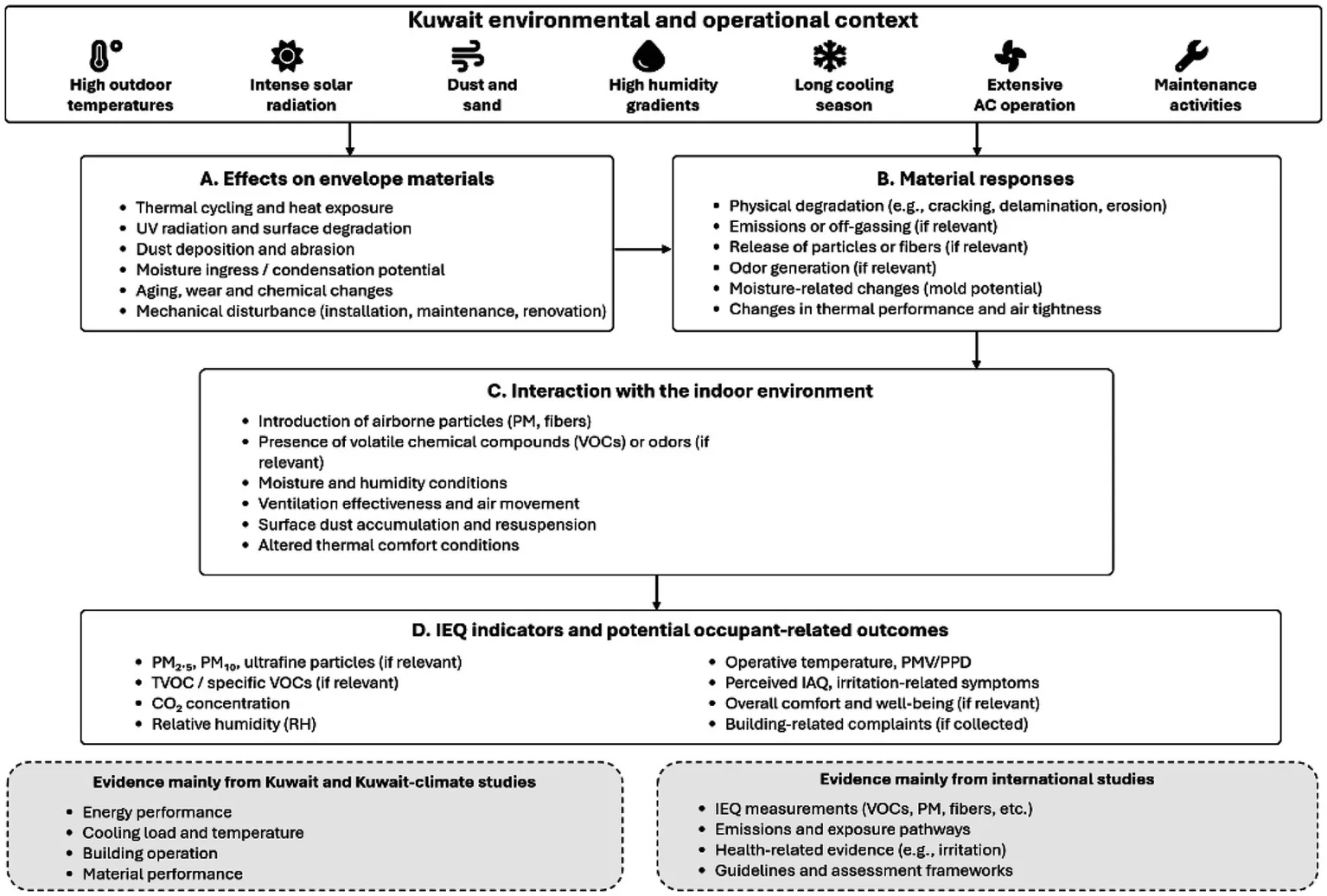
Conceptual framework showing how Kuwait conditions affect envelope materials and how these materials may interact with the indoor environment and occupants, designed using AI.

## 2 Methodology

### 2.1 Review design

This study was conducted as a narrative review because the available Kuwait-related evidence is methodologically heterogeneous, encompassing numerical simulations, field measurements, portable-cabin experiments, material-performance studies, case studies, conference publications, and international reviews. This heterogeneity limits direct quantitative pooling across studies; therefore, the review was designed to provide a structured qualitative synthesis of material categories, environmental and operational stressors, indoor environmental indicators, and potential exposure pathways.

### 2.2 Kuwait-focused literature search

Scopus was used as the primary bibliographic database to identify Kuwait-based and Kuwait-climate studies. The search combined terms related to Kuwait and buildings with terms including thermal insulation, building envelope, phase change materials (PCMs), Bio-PCMs, polyurethane panels, expanded polystyrene (EPS), rockwool, cool roofs, reflective coatings, window films, smart windows, glazing, and walling materials. Reference lists of relevant papers were additionally screened, and DOI information was checked to verify publication details. The final literature search was conducted on April 2026.

### 2.3 Eligibility criteria

The selected Kuwait-based studies were screened according to their relevance to building-envelope or insulation materials, cooling-load reduction, thermal performance, energy efficiency, indoor comfort, or envelope operation. Studies mainly related to oil, petroleum applications, airport runway materials, outdoor pollution without a building-envelope link, or unrelated industrial topics were excluded.

Peer-reviewed journal articles and relevant conference proceedings were eligible for inclusion when they addressed building-envelope or insulation materials under Kuwait-specific or Kuwait-climate conditions. Review articles were used primarily to support broader material, indoor environmental, and public-health interpretation rather than as primary Kuwait-specific evidence. No publication-date restriction was applied. Only English-language publications were considered. Duplicate records and studies without sufficient relevance to building-envelope materials or their thermal, operational, indoor environmental, or occupant-related performance were excluded.

### 2.4 International indoor environmental and public-health evidence

Because the identified Kuwait literature rarely included direct measurements of material-related indoor chemical indicators, particulate matter, fibers, dampness-related exposure, or occupant-health outcomes, a complementary international evidence stream was used to support interpretation of potential indoor environmental and public-health pathways. This literature addresses indoor air quality, VOCs and aldehydes, odor, dust, fibers, particulate matter, moisture and dampness, ventilation, occupant comfort, material aging, and exposure assessment. International evidence was used to identify plausible pathways and appropriate measurement indicators rather than to infer Kuwait-specific exposure levels or health outcomes.

### 2.5 Evidence classification and indicator-selection criteria

To account for methodological heterogeneity, Kuwait-focused studies were classified as numerical simulation, experimental/prototype or portable-cabin studies, occupied-building field studies, and mixed field simulation studies. For each study, when available, season or climatic input, building type, envelope component, layer sequence and thickness, thermal or hygrothermal properties, HVAC operation or setpoint, ventilation or infiltration conditions, and the measured/simulated outcomes were documented. Any missing information was identified as a reporting gap rather than assumed.

Potential indoor environmental and public-health indicators were reported only when (i) a material, product, or disturbance source could be identified, (ii) a physically relevant release and transport pathway to an occupied or work zone could be specified, and (iii) a measurable indicator and an appropriate measurement approach existed. International literature was used to support mechanisms and methods, but not to assign Kuwait-specific exposure levels or health effects. Figure 2 summarizes the study-selection and evidence-classification workflow.

**Figure 2:**
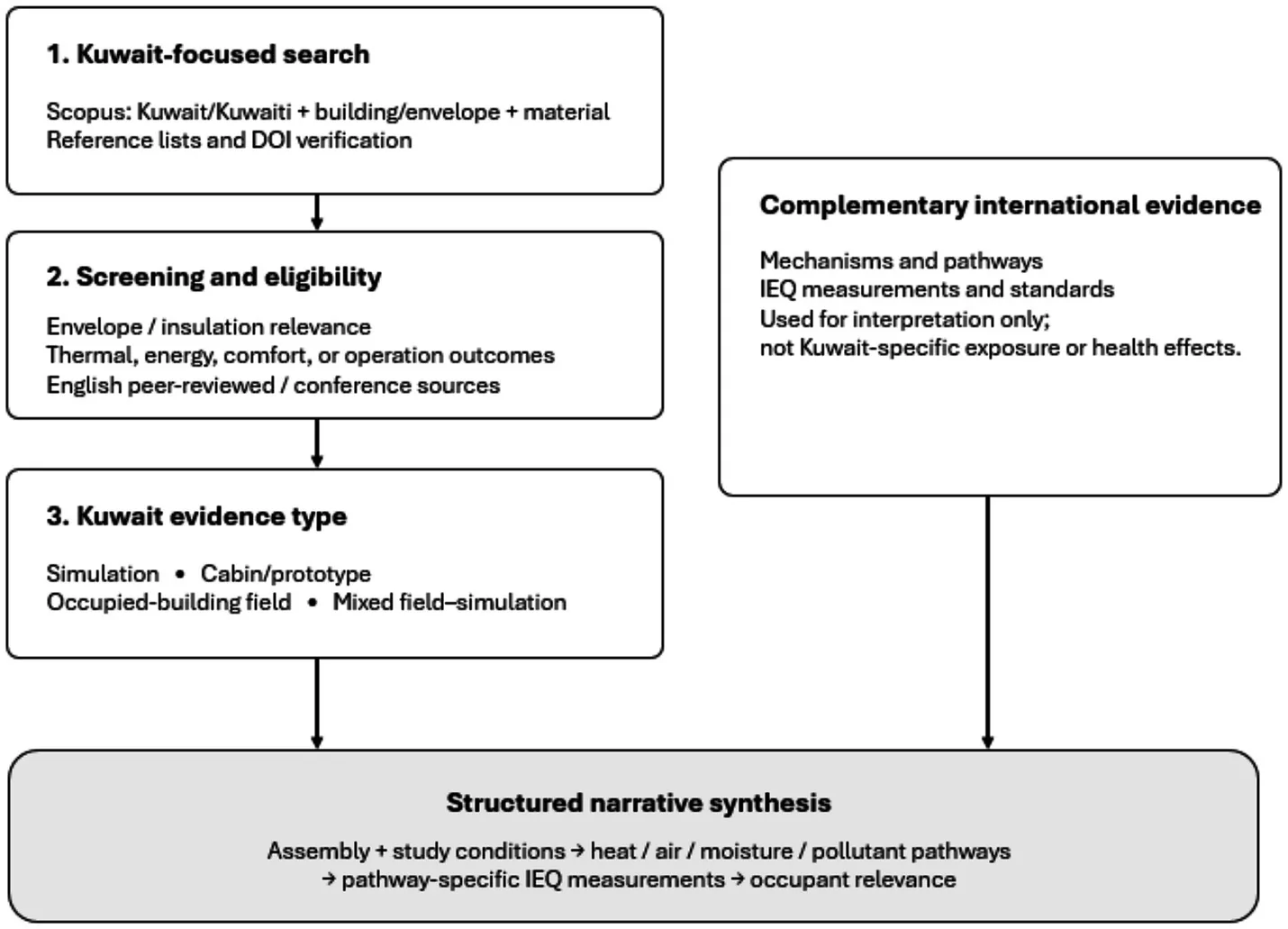
A schema illustrating the study-selection and evidence-classification workflow for this study.

## 3 Thermal insulation and envelope materials identified in Kuwait building studies

The Kuwait-based literature reviewed in this paper identifies several thermal insulation and building-envelope materials that are mainly studied for reducing cooling demand, improving thermal performance, and supporting low-energy building strategies. The most frequently represented materials and systems are phase change materials (PCMs), Bio-PCMs, polyurethane sandwich panels, selected conventional insulation layers, reflective and cool-roof systems, solar-control films, advanced smart-window systems, and thermally efficient walling materials. These studies are valuable because they show which envelope systems are being investigated under Kuwait or Kuwait-like hot-climate conditions. However, their primary focus remains energy performance rather than direct indoor environmental measurements. This section therefore focuses primarily on the material systems identified, their envelope applications, and the thermal or energy-performance evidence reported under Kuwait or Kuwait-climate conditions; indoor environmental and exposure-related implications are examined separately in Section 5.

Figure 3 shows the main building-envelope components and the typical location of insulation and envelope-related materials presented in this review. The schema elaborates the distribution of reviewed systems across roofs, walls, glazing systems, facades, shading elements, joints, and other envelope components. The detailed material categories are then presented in Table 1.

**Figure 3:**
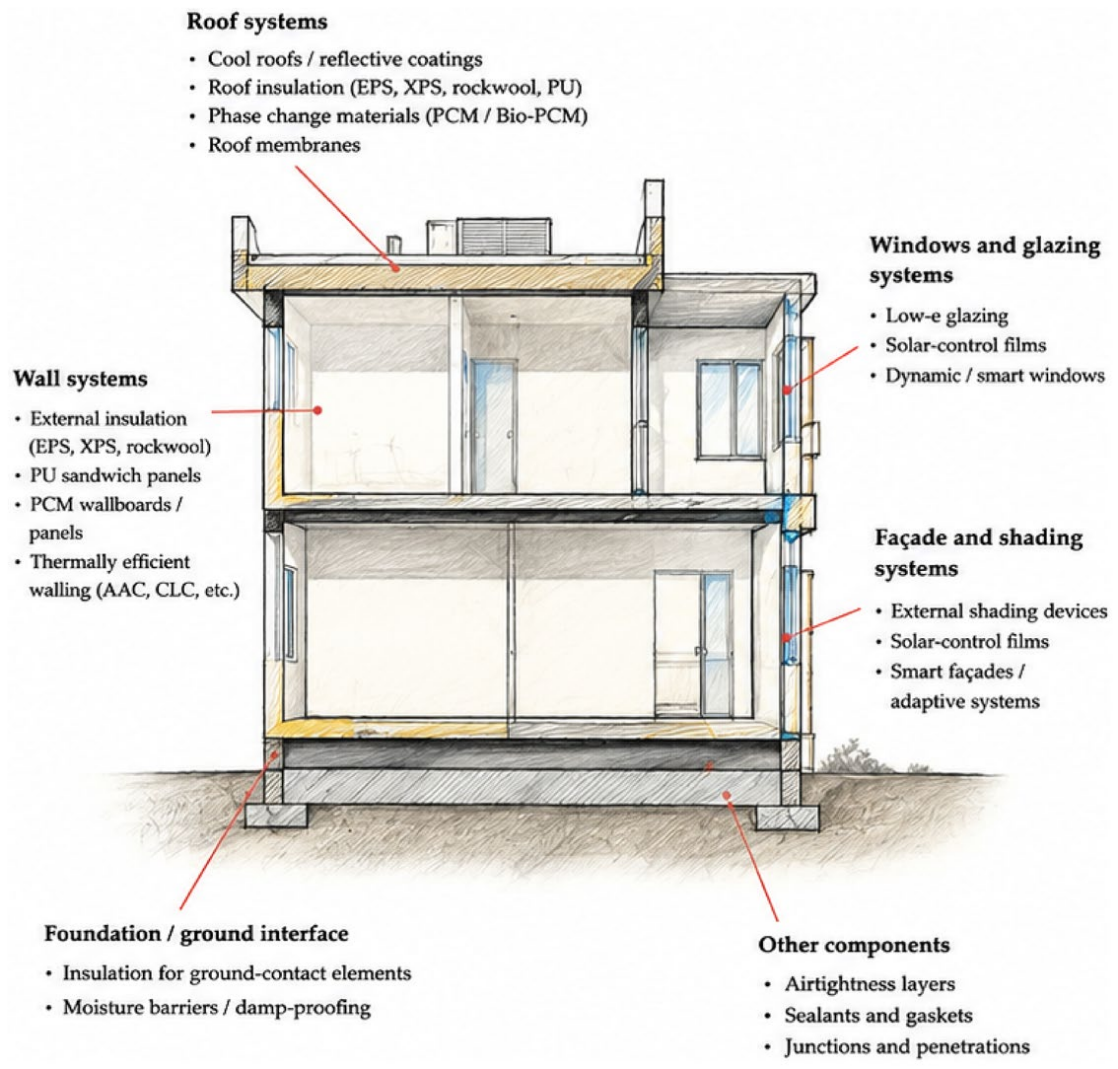
Building envelope components and location of insulation and envelope related materials discussed in this review, designed using AI.

**Table 1:** Thermal insulation and building-envelope materials identified in Kuwait building studies and related indoor environmental considerations.

Material/system category	Materials/systems identified in Kuwait or Kuwait-climate studies	Main building use	Main findings from the reviewed studies	Potential indoor environmental indicators for future assessment	References
Phase change materials and Bio-PCMs	PCM layers; PCM-wallboards; RT-31; RT-35; RT-42; SP-21EK; 24°C PCM; graphite-based PCM; paraffin wax; salt hydrates; eutectic mixtures; organic PCMs; inorganic PCMs; Bio-PCMs	Walls, roofs, ceilings, wallboards, residential envelopes, portable cabins	PCMs and Bio-PCMs are among the most frequently represented envelope-material categories in the Kuwait and Kuwait-climate literature identified in this review. A 20 mm PCM layer was studied in walls and roofs under Kuwait City climate; RT-31, RT-35, and RT-42 were compared. PCM-wallboard simulations for low-rise residential buildings in Kuwait identified a 4 cm wallboard with a 24 °C melting peak as a suitable configuration. Other studies examined PCM-loaded envelopes, graphite-based PCMs, and Bio-PCMs under Kuwait or arid-climate conditions.	Future assessment may consider material formulation, encapsulation stability, leakage potential if panels are damaged, thermal cycling durability, and possible release from organic matrices, additives, or damaged capsules/boards. These aspects were not directly measured in the Kuwait studies.	(11–13, 21, 23–25)
Polyurethane sandwich panels and conventional insulation layers	75 mm polyurethane sandwich panels; 74 mm insulation core with 1 mm galvanized-steel sheets; roof insulation; wall insulation; high-quality insulation; EPS; rockwool	Portable cabins, walls, roofs, residential buildings, heritage envelopes	Polyurethane sandwich panels are clearly identified in Kuwait portable cabin studies. The cabins were used to test cool-roof and Bio-PCM strategies under Kuwait conditions. Green-building and heritage studies also refer to high-quality insulation, EPS, and rockwool in relation to cooling-load reduction and envelope performance.	Future assessment may consider foam-core composition, adhesives, facings, sealants, cutting dust, installation particles, and handling-related fibers in the case of rockwool. The reviewed Kuwait studies identify the materials and energy performance but do not directly assess material-related chemical indicators, fibers, or dust release.	(25–28, 84)
Reflective, faced, cool-roof, and solar-control systems	Cool roof paint; acrylic cool roof paint; aluminium foil roof; aluminium sheet tiles with tilted fins; shingles; reflective/non-reflective roofing systems; solar window films; low-e glazing; double-glass low-e windows	Roofs, façades, windows, office buildings, portable cabins	Reflective and solar-control systems are important in Kuwait because roofs and façades receive strong solar exposure. Finned roof tiles, shingles, cool roof coatings, aluminium foil roofs, reflective roofing systems, window films, and low-e glazing have been examined as energy-saving envelope strategies. The available studies mainly discuss heat-gain reduction, indoor temperature moderation, cooling-load reduction, and energy performance.	Future assessment may consider coating aging, sealants, adhesives, polymer films, reflective facings, and particles generated during installation, maintenance, or replacement. The Kuwait studies focus on thermal and energy performance rather than direct measurement of coating-related chemical or particulate indicators.	(24–27, 29, 30)
Advanced smart façade and window systems	Air-gap smart windows; vacuum glazing; photovoltaic layer; PV-PCM smart windows; AG + PV + PCM + VG systems; ventilated AG + PV + PCM + VG systems; thermochromic smart windows; PNIPAm-based thermochromic hydrogel; PAA/PEG/MXene; Ni–Ti shape memory alloy	Windows, glazed façades, solar-control systems, emerging smart envelopes	Smart-window studies using Kuwait or Kuwait-climate conditions show growing interest in envelope systems that combine insulation, solar control, thermal storage, electricity generation, and ventilation. A sliding smart-window study simulated Kuwait City conditions and compared double air-gap, PV-PCM-vacuum glazing, and ventilated smart-window systems. A thermochromic smart-window study including Kuwait in the simulation examined thermal storage, temperature regulation, and ventilation.	Future assessment may consider long-term performance of polymeric layers, hydrogels, sealants, PV components, nanoparticles, and moving or thermally responsive components under heat and solar exposure. These studies are mainly simulation or prototype studies and do not provide Kuwait field data on indoor environmental performance.	(31, 32)
Thermal walling and envelope materials	Autoclaved aerated concrete (AAC); cellular lightweight concrete (CLC); fired-clay bricks; fly ash bricks; concrete blocks; compressed stabilized earth blocks; calcium silicate blocks; coral stone; replacement wall materials	External walls, model rooms, heritage walls, building-envelope comparisons	These materials are not insulation materials in the strict sense, but they are included because they influence heat transfer and cooling demand. Kuwait-related walling studies compare several wall materials using energy simulations. AAC and CLC were identified as efficient options in terms of cooling-energy performance, while heritage reconstruction studies discuss coral stone and replacement materials in relation to cultural and environmental sustainability.	Future assessment may consider dust generation during cutting, drilling, repair, renovation, or demolition. For these mineral and masonry materials, routine chemical release under normal occupancy is expected to be less central than dust and particle generation during work activities. The reviewed Kuwait studies do not quantify these pathways.	(23, 28)

These indicators represent potential assessment priorities identified from the broader literature and should not be interpreted as measured Kuwait-specific exposures unless explicitly stated.

Table 2 represents Kuwait studies using a consistent assembly and setting level format. NR denotes not reported information.

**Table 2:** A representative summary of Kuwait studies classified by evidence setting and reported envelope-assembly information.

System and reference	Evidence setting	Assembly and layer information	Quantitative information and critical IEQ limitation
PCM in walls/roof (11).	Numerical simulation; Kuwait city climate	20 mm PCM added to vertical walls and roof; RT-31: 27–33 °C, RT-35: 29–36 °C, RT-42: 38–43 °C. Complete substrate layer sequence and vapor/moisture properties: NR.	July model: RT-42 reduced heat gain by 15.37% at the roof and ~13.78% at vertical walls. Thermal model only; no air, moisture, or indoor-exposure measurements.
PCM wallboard (13).	Energy Plus single zone simulation; low-rise air-conditioned residential building	4 cm PCM wallboard; melting peak 24°C; cooling setpoint 24 °C; orientation and window-to-wall ratio varied. Full layer-by-layer hygrothermal properties: NR in this synthesis	Annual cooling-energy savings 4–5%; summer cooling demand/peak-load reduction 5–7%. Simulation evidence; no occupied-building IEQ or exposure measurement.
Portable cabin (25)	Experimental cool-roof tests + TRNSYS Bio-PCM simulation; Kuwait portable cabins	Cabin internal size 2 × 2 × 2.80 m. Walls/roof: 75 mm PU sandwich panel (74 mm insulation + 1 mm galvanized-steel sheets). Simulated roof: 10 mm gypsum board + air gap + 75 mm panel; floor: 18 mm plywood + air gap.	Reported U-values: wall 0.2534 W/m^2^K; roof 0.218 W/m^2^K. Cool roofs tested experimentally; Bio-PCM performance simulated. Vapor permeability/sorption and occupied-building exposure: NR.
Solar window film (29).	Occupied educational offices + calibrated simulation; June–August 2019	Two similar offices with double glazing; one fitted internally with 3 M Neutral 20 window film.	Measured indoor temperature was generally 2–5 °C lower with the film and humidity differed. Direct field thermal/humidity evidence, but no material-attributed VOC, PM, or fiber exposure measurements.

### 3.1 Phase change materials and bio-PCMs

Among the studies identified in this review, phase change materials (PCMs) and Bio-PCMs are probably the most established material group in the Kuwait studies in building envelopes. They are addressed primarily as passive thermal-storage systems that reduce heat gain, cooling demand, and peak cooling load. As an example, Al-Rashed et al. numerically introduced a 20 mm thickness of PCM as an additional layer in roofs and vertical walls in Kuwait City climate conditions and compared the effectiveness of RT-31, RT-35, and RT-42 (11).

PCM wall-board systems have also been studied specifically for air-conditioned low-rise residential building types in Kuwait. Soares et al. simulated wall-board PCM configurations for air-conditioned residential low-rise buildings in Kuwait using EnergyPlus and evaluated parameters such as PCM wall-board thickness, melting-peak temperature, building orientation, and window-to-wall ratio. Among the configurations tested, a 4 cm wall-board with a 24 °C melting peak was identified as the best solution (13).

More recent papers focused on practical issues of PCM selection and application. Sedaghat et al. discussed PCMs based on graphite, inorganic solid-state paraffin, organic and inorganic salt hydrates, eutectic mixtures, and Bio-PCMs, while also emphasizing the importance of aligning PCM melting temperature with real wall and roof surface temperature patterns in Kuwait (21). Another Kuwait-related study by the same authors examined practical issues of PCM placement in portable cabins, where PCM melting temperature had to be aligned with the thermal behavior of the building envelope in Kuwait (22).

In the cases of PCMs and Bio-PCMs, the temperature exposure is not only a mere source of external aging but also the key controlling factor for thermal performance of these materials. Studies carried out in Kuwait reported the influence of melting-point temperature, of material position in layers, of building-envelope position (roof vs. wall), of roof/wall orientation, of air-conditioning setpoint, and of the achievement of material-temperature conditions that allow complete melting and solidification of PCM during daily operation (11, 13). Within studies on cabins, it has been shown that, on top of an expected melting-point value, the selection of appropriate PCM materials should also be based on its ability to operate according to the measured envelope-temperature profile (21, 23).

The usefulness of PCMs was also studied across arid climates such as Kuwait City, where SP-21EK was used in the building envelope. Specifically, in the study by the same researchers, the authors reported that energy savings strongly depend on the scenario and on whether the PCM is effectively activated. For the Kuwait City climate, the study also concluded that PCM selection should be climate-specific (12). Bio-PCMs were also further studied in portable cabin simulations. Specifically, the studied bio-PCM systems reported that energy-saving potential depends strongly on the type of used PCM, on the location in the building, and on the climate (24, 25). Overall, these studies indicate that the behavior of PCM in Kuwait depends strongly on melting temperature, location, thickness, air-conditioning setpoint temperature, and thermal contact with the building envelope.

### 3.2 Polyurethane sandwich panels and conventional insulation layers

Polyurethane sandwich panels are another recurring envelope material category in the Kuwait evidence base, particularly in portable-cabin studies. Specifically, the portable cabin study explains how portable cabins are built using 75 mm sandwich panels and metal frames. It highlights how these cabins were built in Kuwait and how the cool-roof and Bio-PCM building-envelope strategies are being investigated under Kuwait climate and other climatic conditions (25). A finned roof tile study also experimented with portable cabins in Kuwait and explained that the roof was made of 75 mm polyurethane sandwich panels, as were the cabin walls (26).

Other conventional types of insulation materials are identified in the Kuwait-related literature but with less information than in the other two cases (PCMs, sandwich panels). Specifically, green-building work highlighted how the HVAC cooling load of a converted green residential building was reduced by installing high-quality insulation material, an efficient duct system, and using double-glass low-e windows. In the same work, the researchers also reported the reduction in VOC caused by using an eco-friendly paint as replacement for conventional paint. The study is one of the few Kuwait-related studies that explicitly mention the reduction in VOC in the building-material context (27). In heritage reconstruction work, thermal insulation layers were considered in the construction of reconstructed roofs and walls. Specifically, insulation materials included EPS and rockwool. Compatibility with coral stone wall and moisture behavior were also addressed (28).

### 3.3 Reflective, faced, cool-roof, and solar-control systems

Reflective and solar-control building-envelope systems are particularly relevant in Kuwait, where high solar exposure falls on roofs, façades, and windows. Many Kuwait-related studies examine these envelope systems as passive approaches to reduce solar heat gain and the resulting cooling demand. Specifically, the finned roof tile study proposed aluminium sheet tiles with tilted fins as a passive building-envelope cooling strategy. Moreover, they stated that cool roof should not be considered a replacement for roof insulation, since roof insulations directly reduce heat gain and heat loss (26).

In the portable cabin building-envelope Bio-PCM and cool-roof study, the researchers tested experimentally cool-roof techniques based on shingles and finned metal cool roof and simulated Bio-PCM applications in TRNSYS. Specifically, in the tested configuration, Bio-PCMs showed a stronger energy-saving potential than the tested cool roofs (25). In a broader cool-roof and Bio-PCM study, cool roof paint, aluminium foil roofs, shingles, built-up roofs, modified bitumen systems, spray polyurethane foam, and metal roof concepts were also discussed, although these were, in part, presented only as background information or comparative roofing technologies rather than materials tested specifically in Kuwait (24).

Solar window films were also experimentally studied in Kuwait office buildings. Specifically, Sedagat et al. examined two similar offices in a Kuwait educational building with double-glazed windows, one fitted with 3 M Neutral 20 window film, during June–August 2019. The office with window film generally showed indoor temperatures 2–5 °C lower than the comparable office with bare double glazing, while humidity also differed (29). Low-e glazing also appears in green-building constructions, where double-glass low-e windows contributed to the lowering of the HVAC cooling load in combination with other building-envelope measures (27). Reflective and non-reflective roof systems with and without PCM were discussed as well under Kuwaiti climate conditions in the Gulf Conference proceedings (30).

### 3.4 Advanced smart façade and window systems

Advanced smart-window systems constitute an emerging category in the reviewed Kuwait-related climate studies. These systems are not conventional insulation products but are still of interest since they have features that combine thermal insulation, solar control, heat storage, electricity generation, and sometimes even ventilation. Specifically, Ahmed et al. proposed sliding smart windows that integrate air gaps, phase change material, photovoltaic layers, and vacuum glazing. Their simulations were conducted for Kuwait city meteorological conditions, and the base case of the vacuum-glazing-integrated designs showed much lower solar heat gain than the double air-gap base case (31).

Yu et al. introduced a thermochromic smart window that integrates a PNIPAm-based thermochromic hydrogel, PAA/PEG/MXene nanoparticles, and Ni–Ti a shape memory alloy components to provide thermal storage, temperature regulation, and venting. Specifically, Kuwait was included among the simulated climates, and the study reports a reduced annual HVAC energy consumption in Kuwait compared with common glass (32).

These systems are promising from an energy-performance side, but the reviewed studies are primarily simulation- or prototype-oriented.

### 3.5 Thermal walling and envelope materials

Finally, there is a group of thermal walling and envelope materials, such as autoclaved aerated concrete, cellular lightweight concrete, fired-clay bricks, fly ash bricks, concrete blocks, compressed stabilized and unstabilized earth blocks, calcium silicate blocks, coral stone, and replacement wall materials. These materials are not insulation materials in the strict sense, but they influence thermal mass, heat transfer (33), and cooling demand, and therefore relate to the broader envelope perspective.

A Kuwait-related walling material study compared a variety of walling systems across climatic conditions and reported that AAC and CLC reduce the cooling-energy needs compared with other wall materials (A (23).). The heritage reconstruction work on the Khaz’al Diwan also highlights the importance of material selection in Kuwait, especially where coral stone, replacement materials, roof insulation, and wall insulating materials must be considered together with cultural and environmental requirements (28).

### 3.6 Critical synthesis of assembly evidence

Within the available literature, the strongest evidence concerns heat transfer and cooling performance, whereas complete layer-by-layer assembly descriptions and coupled hygrothermal properties are inconsistently reported. Thus, direct comparison is limited since thermal resistance, vapor control, airtightness, interfaces, and material condition may influence performance even if nominal insulation products are similar. Table 2 reports only assembly information clearly provided by the source.

Additionally, the evidence base includes numerical models, portable-cabin/prototype tests, and occupied building measurements where each has benefits and inconvenients. Simulations are useful for parameter sensitivity and design screening but does not support indoor exposure. Cabin studies provide controlled envelope data that does not necessarily represent occupied buildings; and the small number of occupied-building studies provide the most direct indoor environmental context.

## 4 Kuwait-specific climate and operational stressors affecting envelope-material behavior

The performance of thermal insulation and building-envelope materials in Kuwait cannot be fully understood from their nominal thermal properties alone. The same material may behave differently depending on solar exposure, surface temperature, air-conditioning operation, humidity gradients, air movement, installation quality, maintenance history, and aging. These aspects are particularly important in Kuwait, where cooling demand dominates building energy use and where envelope systems are usually assessed through cooling-load reduction and heat-gain control (3, 4).

### 4.1 Temperature and solar exposure

For roof, façade, and window systems, as well as for coatings, sealants, reflective layers, and outdoor insulation, the temperature and the solar irradiance levels outdoors are highly important exposure conditions in Kuwait. The Kuwait Meteorological Center reports average maximum temperatures of 42–46 °C during the hot summer period and also identifies humid summer intervals, indicating that extreme heat and humidity episodes should be considered separately (34). These factors contribute to raising surface temperature, expedite surface aging, and potentially influence the performance of façade components over time. Their effects are especially critical for building-envelope technologies where operating performance is dependent on temperature such as smart-window assemblies, solar-control films, thermochromic materials, Bio-PCMs, and PCMs (29, 32).

At the envelope scale (11), reported modeled July heat-gain reductions of 15.37% for the roof and approximately 13.78% for vertical walls by adding a 20 mm RT-42 PCM layer.

Smart-window layers, solar-control films, reflective roofs, cool-roof coatings, or façade aluminum facings are typically deployed to reduce solar heat gain. In turn, the long-term thermal performance of these materials may be affected by replacement cycle, surface cleaning, surface soiling, film stability, coating adhesion, loss of reflectance, or dust deposition in indoor outdoor conditions (24–26). For envelope systems, the interpretation of thermal performance should not be restricted to a property but should also address the possibility of a change over time due to maintenance operation, heat exposition, solar exposure, or dust accumulation. For coatings, solar-control films, reflective facings, and related envelope layers, long-term assessment should therefore also consider changes in adhesion, surface condition, optical performance, film stability, and material integrity resulting from repeated heat, solar exposure, dust accumulation, cleaning, and aging.

Advanced smart-window and façade systems may require particular attention to long-term material stability because they combine polymeric layers, sealants, photovoltaic elements, PCMs, hydrogels, thermochromic components, and other temperature-responsive materials. Although available Kuwait-climate simulations indicate potential reductions in solar heat gain and cooling demand, field validation is still needed to determine whether prolonged heat, intense solar exposure, thermal cycling, and aging affect sealant integrity, polymer stability, optical performance, and long-term system durability (31, 32).

### 4.2 Humidity, air-conditioning, and moisture movement

Kuwait is defined as a hot-arid environment. However, in certain conditions, buildings are operated under high air-conditioning levels for long time intervals. The indoor environment may therefore result in a high indoor–outdoor temperature difference across walls, roofs, windows, or insulation layers. These conditions may affect heat transfer, vapor movement, drying potential, and joint or cavity operation; especially if indoor air is kept at low temperature and external surfaces are exposed to high solar load conditions (4, 13).

Moisture movement is particularly relevant in multi-layer façades, joints and sealants, and walls or roofs containing insulation layers or cavities. Wall and roof insulation, for example, may be affected by the hygrothermal conditions, the environment surrounding the walls or roof (35, 36), or the compatibility of traditional or replacement material, insulation layer, roof assembly, and environmental conditions (28) for the case of heritage reconstruction work.

For modern building-envelope systems, moisture behavior may also be affected by multi-layer systems and their components such as the position of insulation within the assembly, adhesives, cavities, vapor-resistant layers, or facings.

Energy simulations generally assume stable material properties and intact envelope assemblies over time; however, actual building conditions may deviate from these assumptions. In practical buildings, condensate problems within HVAC systems or roof leakage, failed sealants, cracks, or thermal bridges, defects in workmanship, or operational modifications of materials over time (e.g., material degradation, biological or chemical material failure) may influence the behavior of envelope materials (13). In this respect, the ability of envelope materials to dry, as well as their durability against moisture and long-term building-envelope performance, may be influenced by the movement of moisture. For this reason, in building systems and materials for Kuwait, moisture movement should be interpreted as an envelope component operational condition rather than merely as a property. The indoor environmental interpretation of moisture-related envelopes is discussed further in Section 5.

### 4.3 Ventilation, airtightness, and air movement

The way indoor spaces interact with building-envelope assemblies is also affected by air movement or airtightness and is generally identified as ventilation (e.g., through façade joints, ceiling voids, service penetrations, joints, and cavities). In the case of highly air-conditioned buildings, windows may remain closed for extended time intervals and the indoor air condition may be mainly determined by air filtration, recirculation, infiltration, or mechanical ventilation operation. The way air moves through cavities, façades, or service penetrations, ceiling voids, and joints may also influence the way envelope-related indicators move or dilute into indoor environments.

Studied Kuwaiti ventilation and cooling strategies indicate relevance for indoor air quality conditions, thermal comfort, and energy use when evaluating passive evaporative cooling and ventilation conditions (37), for instance, in wind-catcher studies. In turn, this is relevant to envelope material assessment since the behavior of materials cannot be dissociated from the way air moves across or within the building envelope.

### 4.4 Material aging and weathering

Aging is also important for exposed envelope systems such as cool roofs, solar-control films, coatings, sealants, façades, and smart-window assemblies. Heat, ultraviolet exposure, dust accumulation, cleaning operations, and mechanical damage may affect surface properties, airtightness, reflectance, adhesion, encapsulation, and replacement needs. For PCMs, aging may influence phase-change behavior, encapsulation stability, leak resistance, and thermal-cycling reliability (12, 22).

### 4.5 Installation, maintenance, aging, renovation, and replacement

The behavior of envelope materials may differ between normal use, installation, maintenance, renovation, and replacement. During normal occupancy, many insulation and envelope materials are concealed behind finishes, facings, membranes, boards, panels, or external layers. During installation, repair, cutting, drilling, sanding, removal, or replacement, the same materials may be physically disturbed. Therefore, the building stage should be clearly identified when evaluating material behavior and envelope performance (38–40).

Polyurethane sandwich panels, EPS, rockwool, mineral wool, and other insulation systems require a distinction between intact in-service conditions and disturbed conditions. For foam-based products, relevant aspects may include exposed edges, damaged facings, joints, adhesives, sealants, and cutting quality. For mineral-fiber insulation, handling, access, repair, or removal conditions are more relevant than normal concealed use. For walling and masonry materials such as AAC, CLC, concrete blocks, fired-clay bricks, fly ash bricks, and replacement heritage materials, material disturbance during cutting, drilling, renovation, or demolition is more relevant than routine occupancy.

Where Kuwait studies did not measure aging, emissions, or moisture damage directly, these mechanisms are treated here as testable hypotheses supported by broader literature rather than as observed Kuwait outcomes.

## 5 Indoor environmental exposure pathways and public-health relevance

The Kuwait literature identified in this review primarily evaluates insulation and building-envelope systems through thermal and energy-performance indicators. Direct Kuwait evidence concerning occupant exposure, material-related indoor concentrations, and health outcomes remains substantially more limited. Therefore, this section uses international evidence to examine measurable indoor environmental pathways that may be relevant to Kuwait, including chemical indicators, particulate matter and fibers, moisture-related conditions, ventilation, thermal comfort, and perceived indoor air quality. These pathways are discussed as priorities for measurement and future investigation rather than as evidence that particular envelope materials cause adverse health outcomes in Kuwait.

To avoid confusion between distinct processes, four separate transfer pathways are considered: heat, air, moisture, and pollutants. Heat moves primarily by conduction and radiation and, for PCMs, phase change; air moves through intentional ventilation and unintended leakage; moisture moves by vapor diffusion, air transport, capillary or liquid leakage, and condensation; and pollutants require a source or generation mechanism followed by transport to the occupied zone. Table 3 presents the corresponding evidence and measurements. Consequently, the association of VOCs, particles, or fibers release from a material with the existence of the material in an envelope is not sufficient. If measurable transport pathway is not documented, the indicator is treated only as a future assessment priority.

**Table 3:** Distinct envelope-to-indoor transfer pathways and corresponding measurement logic.

Pathway	Mechanism and envelope-to-indoor route	Key measurements	Interpretation/attribution rule
Heat	Conduction, radiation, thermal bridging, glazing transmission, and PCM phase change through the assembly.	Layer thickness/properties; surface and air temperature; operative temperature; heat flux.	Establishes thermal performance and comfort relevance; it does not by itself demonstrate pollutant exposure.
Air	Mechanical or natural ventilation and unintended leakage/infiltration through joints, cracks, penetrations, cavities, doors, and windows.	Ventilation rate/airflow; pressure; airtightness; window/door status; filtration and HVAC operation.	Defines dilution and transport conditions and is necessary to interpret indoor/outdoor or source-zone concentration differences.
Moisture	Vapor diffusion, air-carried moisture, capillary or liquid leakage, and condensation within or across layers.	Indoor/outdoor RH and temperature; dew-point margin; surface temperature; material moisture; condensation/dampness history.	Moisture is not equivalent to mold; microbial relevance requires persistent moisture plus a susceptible substrate and suitable conditions.
Pollutants	Emission/off-gassing or physical generation after disturbance, followed by air movement from an exposed surface, cavity, or work zone to occupied air.	Targeted VOC/carbonyl sampling; PM₂.₅/PM₁₀; fiber-specific sampling; paired source/occupied-zone or indoor/outdoor measurements.	Attribute VOCs, particles, or fibers to an envelope material only when source + release mechanism + transport route + measured indoor signal are demonstrated.

### 5.1 Chemical exposure indicators and odor perception

Most studies on material-related indoor chemistry are described using the general indicator of total volatile organic compounds, individual VOCs (some of them) and aldehydes, semi-volatile organic compounds, and odor perception. Each of these indicators can be considered relevant to some of the following envelope-related indoor materials and products: adhesive, sealant, coating, polymeric film, foam insulation, binder, PCM matrix, encapsulated material, and smart window components. However, not only the material category, but also product formulation, exposed surface area to be considered, stage of curing, environmental temperature, humidity, air movement and ventilation, covering materials and layers, and product aging can affect the emission behavior (15, 19, 20). Additional studies that are focused on related review papers and modeling also show that indoor residential parameters, emission prediction, indoor simulation, as well as humidity change can affect interpretation of VOC and SVOC indicators (41–44).

Most relevant factors are temperature and humidity, since they can affect release and perception of volatile compounds from materials and products. This is particularly relevant to the Kuwait building context, where exposure conditions of envelope materials may be associated with high surface temperatures, intense solar irradiation exposure, and lengthy air-conditioning periods. In this regard, a review focusing on effects of temperature and humidity on VOC emission behaviors of building materials showed that these two environmental variables, temperature and humidity, can significantly modify VOC emission behaviors from building materials as well, justifying the need to also report indoor temperature, relative humidity, and ventilation conditions in conjunction with any indoor chemical measurement (20).

Aldehydes, and formaldehyde in particular, are particularly often discussed in indoor material-emission studies. For example, formaldehyde, either internally or externally generated, is a Volatile Organic Compound (VOC) that can reach indoor environments both through direct production and through the degradation of some VOCs, and, among the indoor sources of formaldehyde, one can identify furniture products, building materials, and household products. The level of indoor formaldehyde concentration depends not only on the volatile release level of indoor sources, but also on the level of air exchange and on other factors, such as coverings, as well as the temporal emission patterns of indoor sources (45). Reviews focusing on wood-based panels also showed that product formulation, binder type, production process method, and possible mitigation strategies strongly influence formaldehyde-related product performance (46, 47). Although wood-based panels are not the focus of the present review paper, such studies are still relevant because it is a tool to demonstrate the importance of material formulation and standardized testing when assessing indoor chemical indicators.

With regard to insulation materials, the mineral wool deserves some particular attention, since its indoor chemical profile could be related not only to the minerals fibers themselves, but also to binder formulation. A review focusing on low-formaldehyde binders for mineral wool insulation was reported, which shows recent efforts to reduce or replace the formaldehyde-based binder (48), and evidence that mineral wool products should not necessarily be treated as a chemically uniform category. This aspect is relevant to future Kuwait assessments where rockwool or mineral wool insulation may be used for roof, wall, or retrofit applications.

A similar indicators-based approach to polyurethane-based systems, EPS, coatings, sealants, films, and materials and PCM-containing assemblies may also be considered. The proper interpretation is not that these products necessarily affect indoor air quality to a negative extent, but that their chemical profile and emission behavior should be evaluated under realistic use and environmental conditions, particularly after the building installation, during early occupancy, under higher-than-nominal temperature, and after possible aging. Regarding PCM and Bio-PCM-related applications, the current literature used in this review focuses primarily on indoor chemical indicators, and limited direct evidence is provided. Future work in this topic may consider encapsulation stability, potential leakage, odor perception, and volatile indoor chemical indicators during thermal cycling. For polyurethane sandwich panels and EPS systems, Kuwait-specific studies do not provide direct measurements of material-related chemical indicators or indoor concentrations; therefore, chemical assessment should remain framed as a future research need, particularly where foam cores, adhesives, sealants, facings, or exposed edges may be relevant. The same principle applies to solar-control films, coatings, adhesives, sealants, and reflective envelope products: the reviewed Kuwait studies primarily assess thermal or energy performance and do not provide direct Kuwait-specific measurements of material-related indoor chemical concentrations.

Finally, one could discuss the aspect of odor perception as an indoor environmental indicator, and not just as an informal complaint. Odors may affect a perceived air quality, even when it is not possible to identify one or a few compounds that are responsible for the perception. In this regard, the combination of chemical measurements can be completed with odor intensity, odor acceptability, or perceived freshness assessment. A review on building materials and literature related to low VOC emissions for indoor quality and certification in green buildings also shows that indoor air quality evaluation in buildings often relies not on a single chemical indoor indicator alone, but rather on the combination of material selection, ventilation, and low-emitting product criteria (49). This is consistent with the green-building and low-energy-building literature, where tighter envelopes, passive houses, indoor temperature variations, and energy retrofits are commonly discussed not separately, but together with IAQ, comfort, and occupant-related outcomes (50–54).

### 5.2 Particulate matter, dust, and fiber exposure pathways

Dust, fibers, and particulate indicators are especially relevant during material disturbance, installation, renovation, maintenance, or demolition. For insulation and envelope materials, particulate indicators may include PM₂.₅, PM₁₀, inhalable dust, respirable dust, airborne fibers, settled dust, foam particles, and cementitious or mineral particles. Ultrafine particles may also be considered when the research question requires finer particle-size characterization (55). These indicators are not equally relevant for all materials. Mineral wool and rockwool are mainly associated with fibers and dust during handling or disturbance, foam insulation may generate particles during cutting or demolition, and walling materials such as AAC, CLC, concrete blocks, and masonry products may generate mineral dust during drilling or renovation. For polyurethane panels, EPS, rockwool, and mineral-wool insulation, particle or fiber release is likely to be most relevant during cutting, installation, maintenance, removal, or renovation rather than during normal concealed use; however, these pathways have not been directly quantified in the reviewed Kuwait studies.

Notably, evidence from occupied building in Kuwait concerns the infiltration of outdoor particles rather than the release of particles from envelope materials. A previous study investigated indoor and outdoor particulate matters from 10 residences in Kuwait between September 2017 and March 2018 (56). A 20–30% and 40–60% particle penetration efficiencies were reported during dust storms and non-dust storm periods, respectively. These findings show a measurable outdoor to indoor particle pathway, but they do not provide evidence that the particles are originated from building-envelope materials.

For mineral and masonry envelope materials, including AAC, CLC, concrete blocks, fired-clay bricks, fly-ash bricks, and related walling products, particulate exposure is likely to be more relevant during cutting, drilling, repair, renovation, or demolition than during routine occupancy. These activities may generate mineral or cementitious dust, but the reviewed Kuwait-specific studies do not quantify particle concentrations, particle-size distributions, or transfer from work zones into occupied indoor spaces. These materials should therefore be considered primarily within construction-, renovation-, and maintenance-stage particulate assessment rather than as routine indoor emission sources.

Construction-dust reviews show that construction and renovation activities can generate significant particulate matter, and that exposure depends strongly on the activity type, dust-control practices, ventilation, work duration, and particle size fraction (39, 57, 58). These findings are relevant to envelope retrofits because cutting, drilling, sanding, demolition, and cleanup can create short-term particulate peaks that are not representative of normal occupancy.

Fibrous insulation materials require a more specific approach than general particulate matter. A review of insulation fiber deposition in airways shows that fiber behavior depends on fiber dimensions, deposition mechanisms, and exposure conditions (38). Another literature review and quantitative synthesis on man-made vitreous fibers in residential and commercial buildings indicates that exposure assessment depends strongly on the sampling and analytical methods used, including differences between phase-contrast optical microscopy and electron microscopy methods (59).

For the Kuwait material context, these findings suggest that dust and fiber assessment should be stage-specific. In normal occupied conditions, insulation materials are often concealed behind finishes, facings, or panels. During installation, maintenance, ceiling access, renovation, or demolition, the same materials may be disturbed and become more relevant as particle or fiber sources. Therefore, future studies should distinguish between normal occupancy, maintenance activities, and renovation-stage conditions. This distinction is especially important for polyurethane sandwich panels, EPS, rockwool, mineral wool, walling materials, and smart façade systems that may require cutting, replacement, or repair.

Particulate assessment should also consider settled dust. Settled dust can accumulate on surfaces, in ceiling voids, near return-air grilles, or around work zones, and may provide useful information on material disturbance. In future Kuwait studies, real-time particle monitoring could be combined with gravimetric sampling, settled-dust collection, and fiber-specific microscopy where fibrous insulation is involved. Such an approach would allow dust and particulate indicators to be interpreted according to material type, work activity, ventilation condition, and building stage.

### 5.3 Moisture, dampness and microbial indicators

Moisture-related indicators are relevant to indoor environmental quality when water vapor, condensation, dampness, wet materials, or persistent high humidity affect the building envelope or indoor surfaces. Moisture should not be treated as an automatic indication of microbial development. Instead, microbial development should be discussed only when moisture, suitable substrates, and favorable environmental conditions are present. Reviews and guidelines on dampness, measured moisture, and selected indoor pollutants further support the relevance of moisture indicators to indoor environmental interpretation (18, 60–62).

The literature on moisture-sensitive roofing and adhesive systems shows that moisture can affect material performance, adhesion, durability, and service life. Case studies of adhesive and rigid insulation board failures in low-slope roofing assemblies demonstrate that moisture can influence roofing assemblies and insulation-board performance (63). Similarly, moisture-sensitive adhesive failures show that moisture can interact with material formulation and installation conditions (64). These examples are useful because they show that moisture is not only a biological issue, but also a material-performance and durability issue.

Hygrothermal studies also indicate that moisture behavior can vary locally within roof and wall assemblies. A study on roofs with above-rafter thermal insulation emphasized the value of hygrothermal assessment in specific construction details, such as eave overhangs, rather than relying only on whole-assembly assumptions (65). Another study on water vapor and air permeability highlights that vapor movement and air movement are related but distinct transport mechanisms (66). Additional hygrothermal studies on insulated envelopes, post-retrofit housing, internally insulated historic walls, mineral-wool insulation, and highly insulated wall systems further support the need to evaluate moisture and thermal behavior as coupled processes (67–71). This distinction is useful for Section 4 and Section 5 because condensation risk, drying capacity, and air leakage should not be treated as the same process.

Microbial development should be described cautiously and conditionally. A study on the biological susceptibility of earth plasters showed that relative humidity influences fungal growth on susceptible materials (72). This supports the general principle that microbial indicators are relevant when moisture conditions and material susceptibility coincide. It does not imply that all insulation or wall assemblies will support microbial development.

For Kuwait buildings, moisture-related indicators may be relevant because air-conditioning creates indoor–outdoor temperature and vapor gradients, while coastal humidity and intermittent operation may influence condensation potential in some assemblies. Reflective roofs, cool roofs, metal-faced panels, vapor-impermeable layers, mineral wool, EPS, heritage walls, and retrofit systems may all require careful hygrothermal evaluation. Mineral wool storage and pre-installation conditions may also affect performance; one study showed that storage conditions can influence the thermal conductivity of mineral wool in open-space exposure (73).

### 5.4 Ventilation as an exposure modifier

Ventilation is another essential link between material behavior and public-health relevance. Outdoor climate, building operation, ventilation, and indoor exposure are linked together especially where cooling demand is high, as indicated by reviews addressing climate effects on indoor environmental quality (74). Similarly, literature on passive ventilation systems and windcatchers emphasizes that energy efficiency, thermal comfort, and indoor air quality may be influenced simultaneously by ventilation strategies (75). Ventilation standards in residential and non-residential buildings can be informative for future studies reporting ventilation and air-exchange conditions (76, 77).

In envelope-related exposure assessment, ventilation should therefore be treated as an exposure modifier rather than simply as a building-operation variable. Air exchange, recirculation, filtration, infiltration, and airflow through cavities or service penetrations can influence whether material-related chemical or particulate indicators accumulate, dilute, or reach occupied indoor spaces. This distinction is particularly relevant in highly air-conditioned buildings where windows may remain closed for prolonged periods.

### 5.5 Occupant comfort and perceived indoor air quality

Perceived indoor air quality and occupant comfort are relevant because indoor experience can be influenced by building-envelope materials through more than chemical or particulate exposure pathways. Insulation, PCMs, reflective roofs, low-e glazing, solar-control films, and smart windows can affect operative temperature, mean radiant temperature, surface temperatures, thermal stability, glare, perception of daylighting, indoor humidity, and perception of fresh air. It follows that energy performance does not always define the occupant experience.

An integrated view of indoor environmental quality is supported in the literature on construction materials and occupant outcomes. A systematic review on construction materials and occupant outcomes indicates that materials can be related to several indoor environmental dimensions, including physical and psychological responses (19). The literature on green-building certification also supports the tendency to evaluate indoor air quality in terms of combined criteria, including material selection, ventilation, chemical indicators, and comfort-related aspects (49). A review of climate-related effects on indoor environments also supports the need to interpret thermal comfort, ventilation, and indoor air quality together, particularly in climates where cooling demand is high (74). Objective indoor air quality studies and thermal-comfort standards can also serve as useful reference points for combined monitoring of indoor conditions and occupant responses (78, 79).

Perceived indoor air quality may also be affected by odor, stuffiness, air freshness, dryness, thermal discomfort, and adequacy of ventilation. For example, low humidity and air-conditioned office environments may lead to dryness-related discomfort, which occupants may interpret as poor air quality, even when a single pollutant source is not clearly defined. A perspective paper on dry eye symptoms in the office highlights the relevance of indoor environmental conditions to comfort and work performance (80).

This is particularly relevant in Kuwait because buildings are commonly air-conditioned for long time periods. Envelope improvements may reduce cooling demand and stabilize indoor temperatures, but they may also influence perceived freshness, humidity, or daylight conditions. For example, solar-control films and smart glazing may reduce heat gain near façades but can also alter visual perception and daylight availability. Envelope systems with high insulation or high airtightness may improve thermal stability, but ventilation must still be adequate to sustain perceived indoor quality. PCM systems may reduce temperature fluctuations, but occupant comfort still depends on air movement, humidity, odors, and ventilation.

Thermal comfort is also part of the public-health link. In hot climates, envelope systems and insulation are often specified with the primary goal of reducing cooling demand and heat gain but can also influence occupant satisfaction, perception of dryness, humidity, surface temperature or radiant temperature, indoor temperature stability, and other comfort-related indoor conditions. In fact, a scoping review on indoor air quality and thermal conditions in educational buildings shows that performance, wellbeing, thermal conditions, and IAQ are interrelated dimensions of the indoor environment (81). Although these data are not specific to buildings in Kuwait, they support the broader interpretation that occupant-centered indicators should be assessed together with energy-efficient envelope design.

### 5.6 Building stage and exposure context

The installation, maintenance, renovation, and replacement stages should be considered separately from normal occupancy. Many material-related indoor indicators are most relevant during these stages because materials may be cut, drilled, sanded, bonded, coated, removed, or disturbed. The resulting conditions may vary substantially from those in a finished and stabilized building.

The literature on construction dust shows that particulate concentrations are closely related to activity type, work intensity, ventilation, dust suppression, and cleaning practices (39, 57). A review on air pollution in the construction industry also emphasizes that the construction stage can involve multiple air-quality indicators, including dust and activity-related pollutants (40). These references support the need to distinguish between occupant-stage and worker-stage conditions.

For chemical indicators, installation-stage considerations may include adhesives, sealants, coatings, sprayed or liquid-applied products, films, and bonding layers. Early curing or drying periods may produce transient odor or chemical indicators that differ from long-term conditions. This may be relevant for polyurethane sandwich-panel joints, cool-roof coatings, solar-control films, smart-window sealants, and PCM panels that require adhesives or encapsulating systems.

For particulate indicators, installation and renovation stages may be especially relevant for mineral wool and rockwool, EPS, polyurethane panels, cementitious walling materials, and masonry products. Cutting or removing mineral wool may generate fibers, while sawing or drilling foam panels and walling materials may generate dust and particles. Literature on MMVF exposure and insulation fiber deposition supports the need to treat fibrous materials using fiber-specific methods and to distinguish normal indoor conditions from disturbance conditions (38, 59).

Moisture conditions during maintenance stage should also be considered. Failed sealants, roof penetrations, damaged coatings, poorly repaired joints, or HVAC condensate concerns may alter the environmental behavior of an envelope assembly over time. Studies on roofing and adhesive failure show that localized moisture problems can affect assembly performance and durability (63, 64).

For future Kuwait studies, it would be useful to pursue a staged assessment approach. At a minimum, studies could distinguish between: (i) post-installation or first-occupancy conditions, (ii) stabilized routine occupancy, and (iii) maintenance, retrofit or disturbance conditions. This would help separate temporary installation-related indicators from long-term indoor environmental performance.

## 6 Public-health implications and research priorities for Kuwait

The public-health relevance of building-envelope and insulation materials is best considered through indoor exposure pathways rather than material type alone. In Kuwait, this perspective is particularly relevant given that envelope materials might be exposed to repeated maintenance or replacement activities, long cooling periods, dust deposition, thermal cycling, high surface temperatures, and intense solar radiation. These may affect occupant comfort, moisture behavior, particulate release upon disturbance, odor perception, chemical indicators, material aging, or surface condition over time.

From a public-health perspective, most relevant indicators are those that indicate plausible occupant exposure and indoor environmental experience, including PMs fibers, selected VOCs and aldehydes, moisture and dampness, ventilation adequacy thermal conditions, odor perception, and perceived indoor air quality. Such indicators shall be interpreted as part of broader indoor environmental framework rather than as isolated properties of individual construction materials (19).

### 6.1 Key public-health implications

Material aging and moisture behavior are of particularly relevance for envelope systems because insulation or energy retrofit strategies may affect heat, air, and moisture transfer across the assembly. If moisture transfer is not adequately managed, then thermal improvement may be accompanied by localized condensation, material deterioration, or limited drying capacity in specific parts of the envelope. A review focusing on hygrothermal and climatic retrofit strategies supports that energy performance, climatic design, moisture control, and occupant health should be assessed together in building net-zero and retrofit strategies (82). This is of particular relevance for Kuwait because indoor–outdoor vapor and thermal gradients may be particularly strong in air-conditioned interiors that are heavily conditioned.

Changing environmental conditions support the particular relevance of climate-sensitive indoor assessment. The literature on passive envelope strategies and windcatchers also indicates that indoor thermal comfort and air quality should be assessed in combination with occupant wellbeing in dense urban and highly cooled environments (83).

### 6.2 Evidence gaps in Kuwait

The principal evidence gap noticed throughout this review is the limited integration of energy-performance measurements along with direct indoor environmental and occupant-related assessment in Kuwait buildings. The literature provides comparatively stronger evidence on cooling-load reduction, heat-transfer control, and envelope thermal behavior than on long-term material aging, indoor chemical concentrations, particulate or fiber exposure, moisture-related indoor conditions, ventilation-mediated exposure, or occupant responses. Accordingly, future Kuwait-based studies should examine these dimensions in an integrated manner, rather than inferring public-health relevance from energy-performance findings.

### 6.3 Proposed integrated assessment framework

Based on the evidence gaps identified above, future Kuwait studies should adopt an integrated assessment protocol combining material, environmental, ventilation, exposure, and occupant-related measurements. Such an approach would allow thermal and energy performance to be interpreted together with building operation, material condition, indoor environmental quality, and potential exposure pathways.

At the building-operation level, studies should report air movement, airtightness, ventilation strategy, filtration, infiltration, air-conditioning operation mode, indoor temperature, relative humidity, and occupancy conditions alongside material and thermal data. Without this information, it may be difficult to distinguish material-related behavior from operational effects or to compare envelope systems across buildings.

Moisture assessment should extend beyond room-average relative humidity to include surface temperature, dew-point margin, material moisture content, visible dampness, condensation history, drying capacity, and maintenance records. Microbial sampling should be considered when evidence of dampness or persistent moisture is present rather than as a routine first step in all buildings.

Physical measurements should additionally be complemented by occupant feedback. Relevant measurements may include air temperature, radiant temperature, relative humidity, air velocity, carbon dioxide as an indicator of ventilation conditions, surface temperatures, and, where relevant, particulate or chemical indicators. These measurements may be paired with short questionnaires addressing thermal sensation and acceptability, odor intensity, perceived air freshness, dryness, and overall satisfaction. This integrated approach would allow insulation and envelope systems to be evaluated not only for energy savings but also for material performance, exposure conditions, comfort, and perceived indoor environmental quality.

For practical implementation, Table 4 presents the framework as a minimum set of recommended measurements. The selection of parameters shall depend on the pathway and chemical or fiber measurements would be required only if plausible source and transport route exist.

**Table 4:** Practical pathway-based measurement matrix for future Kuwait building-envelope studies.

Domain	Core parameters	Suggested measurement approach	Relevant standard/guidance
Assembly/thermal	Layer sequence and thickness; k/R/U where available; air/surface/operative temperature; mean radiant temperature; air speed; heat flux.	Drawings and product data verified on site; temperature/surface sensors; heat-flux measurement; thermal-environment instruments.	(78, 85)
Air/ventilation	Mechanical ventilation rate; airflow/ACH; pressure; airtightness/infiltration; filtration; window/door and HVAC status; CO₂ as contextual indicator.	Airflow or tracer-based assessment as appropriate; fan pressurization for envelope leakage; continuous operational logging.	(76, 77, 86)
Moisture	Indoor/outdoor RH and temperature; dew-point margin; surface temperature; material moisture; visible dampness/condensation; drying history.	Continuous RH/T logging; surface sensors/infrared inspection; material moisture measurement; maintenance and leak records.	Assembly-specific hygrothermal assessment; report season, exposure, HVAC operation, and coastal/humid periods.
VOC/carbonyl	Selected VOCs and formaldehyde/carbonyls only where a plausible material/source pathway exists.	Sorbent-tube active sampling with thermal desorption GC–MS; DNPH active sampling with HPLC for carbonyls.	(87, 88)
Particles/fibers	PM₂.₅/PM₁₀; airborne fibers where fibrous insulation is disturbed; source-zone and occupied-zone context.	Filter/gravimetric PM measurement supplemented by high-time-resolution instruments; fiber-specific microscopy where relevant.	(89, 90)
Occupant response	Thermal sensation/acceptability; odor; perceived freshness/dryness; perceived IAQ and overall satisfaction.	Short questionnaire paired in time and location with physical measurements.	(78)

At minimum, field studies should report season, building type, orientation, occupancy, HVAC schedule and setpoint, ventilation mode and airflow, filtration, window/door status, and infiltration or airtightness. Occupied buildings should be prioritized when exposure or health relevance is claimed; simulation and portable-cabin studies should remain identified as design/performance evidence. Repeated measurements across hot-dry and humid periods and across post-installation, stable-occupancy, and maintenance/renovation stages would improve attribution.

Four priorities emerge for Kuwait: (1) occupied-building studies pairing envelope characterization with indoor environmental measurements; (2) coupled heat–air–moisture monitoring of complete assemblies; (3) source-to-zone confirmation before attributing chemical, particulate, or fiber indicators to envelope materials; and (4) longitudinal aging and maintenance studies combined with occupant feedback.

### 6.4 Limitations of the review

This review should be interpreted while considering a number of limitations. First, the Kuwait-based evidence base is relatively limited and heterogeneous, encompassing broader building-performance investigations, case studies, field measurements, experimental cabin studies and numerical simulations. As a result, comparison across studies is limited by assessment methods, performance indicators, operating schedules, climatic inputs, and building type and envelope configuration. The review thus focuses on qualitative synthesis rather than pooling of reported outcomes.

Second, direct measurements of occupant exposure, long-term aging, and material-related indoor environmental indicators are relatively limited in the Kuwait-based literature. The available literature is weighted toward thermal and energy performance assessment and less toward direct measurement, although findings are again heterogeneous and do not provide an homogeneous picture. As a consequence, public-health relevance, ventilation, moisture-related conditions, particulate matter or fibers, dust, odor, and chemical indicators are discussed with support mainly from international literature and aim to identify future research needs and assessment pathways rather than inferring Kuwait-specific health or exposure outcomes.

Third, not all material categories are similarly represented in Kuwait-based literature. Conventional insulation materials are studied with lower frequency than emerging smart-envelope systems and passive envelope strategies or PCMs. Also, several advanced technologies remain mainly prototype- or simulation-based. Future Kuwait-based studies combining occupant feedback, indoor environmental measurements or hygrothermal assessment, material characterization, and long-term field monitoring may help fill these evidence gaps and enhance comparison across envelope solutions.

Fourth, this study was conducted as a narrative rather than systematic review; consequently, formal risk-of-bias assessment and quantitative evidence grading were not performed. Scopus served as the principal bibliographic database, supplemented by reference-list screening and DOI verification, and relevant studies indexed exclusively in other databases may therefore not have been captured. These methodological characteristics should be considered when interpreting the completeness of the evidence base.

## 7 Conclusion

This review identified an emerging body of Kuwait-focused research examining thermal insulation and building-envelope systems primarily from the perspective of cooling-load reduction and energy performance. Direct Kuwait evidence linking envelope materials to material-related indoor concentrations, occupant exposure, or health outcomes remains limited. Public-health relevance should therefore be interpreted through measurable pathways and should not be inferred from material type or energy performance alone. The principal contribution of this review is a structured assembly–transport–IEQ–occupant framework that connects Kuwait’s established thermal-energy evidence with explicit heat, air, moisture, and pollutant pathways. Future work should prioritize occupied-building studies that document complete envelope assemblies and operating conditions and use standardized, pathway-specific measurements of ventilation, moisture, thermal conditions, and only where a plausible source-to-indoor route exists, chemical, particulate, or fiber indicators together with occupant feedback.
